# Impact of Prediabetes and Type-2 Diabetes on Outcomes in Patients with COVID-19

**DOI:** 10.1155/2021/5516192

**Published:** 2021-06-16

**Authors:** Jasbir Makker, Haozhe Sun, Harish Patel, Nikhitha Mantri, Maleeha Zahid, Sudharsan Gongati, Sneha Galiveeti, Sharon W. Renner, Sridhar Chilimuri

**Affiliations:** ^1^Department of Medicine, Bronxcare Health System, Affiliated with Icahn School of Medicine at Mount Sinai, Bronx, New York, NY, USA; ^2^Division of Gastroenterology, Bronxcare Health System, Affiliated with Icahn School of Medicine at Mount Sinai, Bronx, New York, NY, USA; ^3^Division of Endocrinology, Bronxcare Health System, Affiliated with Icahn School of Medicine at Mount Sinai, Bronx, New York, NY, USA; ^4^Department of Kinesiology and Health Sciences, Columbus State University, Columbus, Georgia

## Abstract

**Introduction:**

The true impact of prediabetes and type-2 diabetes in patients with COVID-19 remains unknown, with studies thus far providing conflicting evidence.

**Methods:**

This is a single-center retrospective observational study involving 843 hospitalized patients with SARS-CoV-2 infection. Primary outcomes, mortality, and mechanical ventilation use were compared among the three groups: control, prediabetes, and type-2 diabetes. Binomial regression analysis was used to determine predictors of mortality and mechanical ventilation requirement.

**Results:**

Age was a significant predictor of mortality. On stratifying our patients based on their age, older patients aged 55 years and above had no difference in mortality or mechanical ventilation requirement among the three groups of control, prediabetes, and type-2 diabetes. However, among the younger population aged less than 55 years, patients with type-2 diabetes had significantly higher mortality as compared with patients in control and prediabetes groups (27% vs 12.5% vs 9%, *p* 0.025). Additionally, newly diagnosed type-2 diabetes patients demonstrated lower mortality rate in comparison to previously known type-2 diabetes patients (18% vs 40%, *p* 0.005). Outcomes in the prediabetes group were similar to that in the control group. Admission hyperglycemia was associated with higher mortality regardless of diabetes status.

**Conclusion:**

In older patients aged 55 years and above, status of type-2 diabetes does not influence their mortality. However, in younger patients aged less than 55 years, the presence of type-2 diabetes is an important driver of mortality. Newly diagnosed type-2 diabetes, in comparison with previously diagnosed type-2 diabetes, may have better survival. Presence of prediabetes did not affect outcomes in patients with COVID-19 infection.

## 1. Introduction

Severe acute respiratory syndrome coronavirus 2 (SARS-CoV-2), the virus that causes COVID-19, became a public health emergency of international concern soon after the start of the outbreak in Wuhan, China. It has infected more than 157 million individuals and claimed more than 3.2 million lives worldwide so far [1], with an overall mortality rate of 2.08%. Although most of the individuals infected with COVID-19 will present only with minor symptoms, some will present with severe illness in the form of acute respiratory distress syndrome and death. Early reports from China during the initial phase of COVID-19 outbreak demonstrated presence of one or more coexisting illnesses associated with worse outcomes. There was two- to fourfold higher prevalence of type-2 diabetes in COVID-19 patients with increased mortality in these studies [2, 3]. Later similar reports emerged from other parts of the world highlighting the poor outcomes in patients with type-2 diabetes infected with severe COVID-19 infection [4–6]. On the other hand, a large-scale observational Coronavirus SARS-CoV-2 and Diabetes Outcomes (CORONADO) study involving 68 centers in France surprisingly showed no impact of past glucose control in type-2 diabetes patients hospitalized with COVID-19. CORONADO study, instead, recognized hyperglycemia at the time of admission as a predictor of poor outcomes in patient with type-2 diabetes [7].

As the pandemic evolves and claims more lives, not only the true impact of type-2 diabetes in patients infected with COVID-19 remains unknown but pathophysiology underlying the poor outcomes in type-2 diabetes patients with COVID-19 is not fully understood as well. Studies published so far, as mentioned above, have provided conflicting evidence. Additionally, very little is known or studied about outcomes of COVID-19 in patients with prediabetes, who have similar metabolic derangements, though not to the same extent as in type-2 diabetes. There is a complete paucity of data reflecting the effect of these partial metabolic derangements present in prediabetes on outcomes of COVID-19. We conducted this study with two aims in mind:

1. To study the effect of type-2 diabetes on outcomes in COVID-19 patients in our study population which predominantly comprises Latinx and African American patients.
2. To explore the impact of prediabetes on outcomes in COVID-19 patients.

## 2. Methods

This is a single-center retrospective observational study. Electronic medical records for all the 1277 patients aged 18 years and above with COVID-19 RNA confirmatory nasopharyngeal swab consecutively hospitalized at our hospital between March 9, 2020, and June 27, 2020, were reviewed. Demographic data including age, gender, and ethnicity were collected from electronic medical record review. Symptoms at presentation, all the comorbid conditions, medications administered during the hospitalization, imaging study findings, and laboratory parameters at admission were also abstracted. The most recent hemoglobin A1c (HbA1c) lab value for all the patients within preceding one year until the day of admission was noted as well. The study was performed in accordance with the Declaration of Helsinki and was approved by the Institution Review Board (IRB) of Bronx Care Hospital Center.

### 2.1. Patient Selection

#### 2.1.1. Type-2 Diabetes Group

Patients were categorized as “type-2 diabetes group” utilizing both ICD 10 code E 11.9 for type-2 diabetes and HbA1c lab value within preceding one year until the day of admission. If the patient had more than one HbA1c lab value available, then the most recent one was used for the study purpose. HbA1c ≥ 6.5% was considered diagnostic of type-2 diabetes as per the American Diabetes Association criteria.

**Figure 1:**
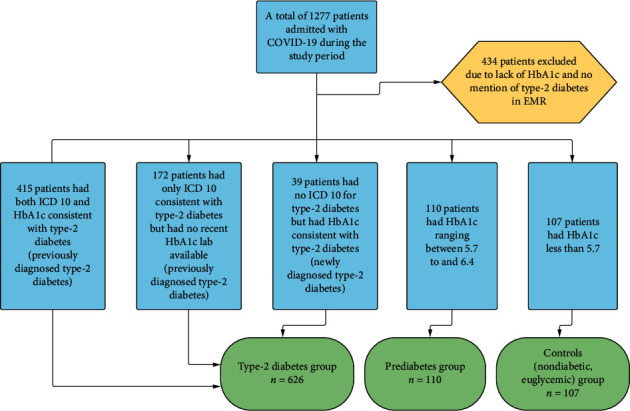
Flow diagram of patient selection.

There were 415 patients who had ICD 10 as well as hemoglobin A1C lab value consistent with the diagnosis of type-2 diabetes. Additionally, 172 patients had no hemoglobin A1C lab available within preceding one year or at admission but were still categorized as type-2 diabetes based on their ICD 10 code. Health care provider notes were also reviewed in detail to confirm the diagnosis of type-2 diabetes in these patients. There were a total of 39 patients who had no prior history of type-2 diabetes and were newly diagnosed as type-2 diabetes at the time of admission. Overall, there were a total of 626 patients in the type-2 diabetes group (Figure 1).

#### 2.1.2. Prediabetes Group

Patients hospitalized with COVID-19 and HbA1c lab value ranging between 5.7 and 6.4 at admission or within preceding one year, whichever was most recent, were classified as “prediabetes group.”

#### 2.1.3. Control (Euglycemic Nondiabetic) Group

Patients hospitalized with COVID-19 and HbA1c lab value less than 5.7 at admission or within preceding one year, whichever was most recent, and no mention of type-2 diabetes in their health record were classified as the “control (nondiabetic or euglycemic) group.”

#### 2.1.4. Primary Outcomes

Primary endpoints were mortality and tracheal intubation for mechanical ventilation.

#### 2.1.5. Statistical Analysis

Statistical analysis was performed with IBM SPSS (Statistical Packages for the Social Sciences) version 19. Frequencies and percentages were reported for categorical variables. Mean and standard deviations were reported for numerical continuous variables. Dichotomous variables were compared by chi-square analysis using the Pearson test, and continuous variables were compared using one-way analysis of variance (ANOVA). Binomial regression analysis was used to find statistically significant predictors of mortality and need for mechanical ventilation. A two-tailed value of <0.05 was considered statistically significant. Kaplan–Meier survival curves were used to compare survival times among those with preexisting diagnosis of diabetes and those with newly diagnosed diabetes.

## 3. Results

The study group with type-2 diabetes comprised a total of 626 patients, whereas the group with prediabetes had 110 patients and control group had 107 patients. Patients in the type-2 diabetes group were older with the mean age 65.4 ± 14 years as compared to 62.2 ± 15 years in the prediabetes group and 58.6 ± 17 years in the control group (p 0.001). Male patients were predominant in all the three groups accounting for 59%, 59%, and 64% in the type-2 diabetes, prediabetes, and control groups, respectively. Patients in the type-2 diabetes group had higher BMI of 30.1 ± 8 kg/m^2^, as compared to 29.8 ± 8 kg/m^2^ in the prediabetes group, and 28.1 ± 8 kg/m^2^ in the control group (p 0.024). Most of the patients across all three groups belonged to Latinx or African American ethnicity. Symptoms present at the time of admission were not significantly different among the three groups. The type-2 diabetes group had significantly higher percentage of patients with hypertension and coronary artery disease. Absolute neutrophil count and C-reactive protein at the time of admission were significantly elevated in the type-2 diabetes group. Tamiflu and hydroxychloroquine were administered in higher percentage of patients in the type-2 diabetes group (Table 1). Thirty-nine percent of patients with type-2 diabetes died due to COVID-19, in comparison to 32% in the prediabetes group and 30% in the control group (p 0.036). Similarly, higher percentage type-2 diabetes patients (40%) required tracheal intubation and mechanical ventilation, in comparison to 31% in the prediabetes as well as the control group (p 0.027).

Using binomial regression analysis, age was found to be a significant predictor of mortality, which is consistent with previously reported data from our institute [8] as well as data on COVID-19 mortality worldwide. Neither the presence of type-2 diabetes nor prediabetes predicted the overall mortality (Table 2). Using binomial regression analysis for mechanical ventilation need, BMI was a significant predictor (Table 3).

Next, we stratified the data based on their age less or more than 55 years into younger and older age groups, respectively, as per United States Census Bureau definition of old age. We compared mortality (Table 4) and mechanical ventilation need (Table 5) between these younger and older age groups among the three categories of control (euglycemic nondiabetic group), prediabetes, and type-2 diabetes. Mortality rates among controls, prediabetes, and type-2 diabetes were not different in the older population aged more than 55 years. However, in the younger population aged less than 55 years, patients with type-2 diabetes had significantly higher mortality (27%) as compared to controls (12.5%) and patients with prediabetes (9%, *p* 0.025). To explain this difference in mortality, we further looked into mean BMI among the three groups in these younger patients aged less than 55 years and found no significant difference in their BMI (mean BMI control group 32.4 ± 8.4 kg/m^2^, prediabetes 34.5 ± 11.5 kg/m^2^, and type-2 diabetes 32.8 ± 111.1 kg/m^2^; *p* 0.664). We also looked at mean blood glucose level at the time of admission in these younger type-2 diabetes patients with higher mortality and found it be higher but not significantly different from those of older type-2 diabetes patients (285 ± 234 mg/dL vs 257 ± 190 mg/dL, *p* 0.1496). Need for mechanical ventilation was not significantly different between younger and older patients across three groups: control, prediabetes, and type-2 diabetes.

We found a total of 39 patients (6.2%) who were newly diagnosed with type-2 diabetes at the time of admission. On comparing these, 39 newly diagnosed type-2 diabetes patients with the remaining 587 patients of previously known type-2 diabetes, they had lower mortality rate (18% vs 40%, *p* 0.005), their mechanical ventilation requirement was also lower, but this was marginally significant (26% vs 41%, *p* 0.06) (Table 6). In our study patients, blood sugar levels at the time of admission for those with previously diagnosed type-2 diabetes were significantly higher from those with newly diagnosed type-2 diabetes (267 ± 206 mg/dL vs 200 ± 90 mg/dL, *p* 0.04). Mean blood sugar levels for patients with prediabetes and control groups at the time of admission were 124 ± 31 mg/dL and 122 ± 45 mg/dL, respectively.

**Figure 2:**
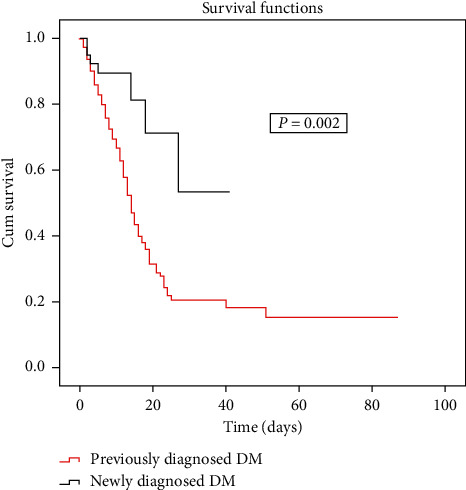
Kaplan–Meier survival curve comparing the two groups of previously diagnosed and newly diagnosed type-2 diabetes.

Using binomial regression analysis for mortality among these patients with type-2 diabetes, having a previously diagnosed type-2 diabetes was a significant predictor of mortality. Age and steroid use were also significant predictors of mortality (Table 7). The Kaplan–Meier curves showed better survival rates among newly diagnosed type-2 diabetes in comparison to previously diagnosed type-2 diabetes patients (p 0.002) (Figure 2).

We also compared mortality among our three groups of controls, prediabetes, and type-2 diabetes stratified into three categories based on their admission glucose level: less than mg/dL, 141 to 180 mg/dL, and more than 180 mg/dL (Table 8). Mortality in all the three groups, controls, prediabetes, and type-2 diabetes, was higher in the individuals with admission blood glucose level more than 180 mg/dL as compared with individuals with admission blood glucose level less than 140 mg/dL.

## 4. Discussion

The results of this study show that age remains the biggest risk factor for mortality in patients hospitalized with COVID-19 infection. In patients with prediabetes who are infected with COVID-19 infection, mortality is no different from the control group. Similarly, in patients with type-2 diabetes who are older than 55 years of age, mortality is similar to the control group. However, in the younger type-2 diabetes patients aged 55 years and less, mortality is significantly higher than the nondiabetic patients in the control group.

Right from beginning of the pandemic, advanced age was found as a predictor of severe COVID-19 infection and mortality [2]. In a meta-analysis involving more than half a million subjects infected with COVID-19 across five countries, mortality rates increased exponentially after age 50 and were highest among the octogenarians [9]. Our study results are consistent with this previously reported data demonstrating higher mortality among older age group. Though the data across the globe consistently show elevated mortality rates among older people, these mortality rates are not similarly elevated across the globe. Thus, additional drivers of increased mortality in these patients with COVID-19, like presence of other comorbid conditions, likely play an important role.

Due to a heavy global burden of type-2 diabetes on public health, several researchers have focused so far on studying its impact on outcomes in patients with COVID-19. Though poor outcomes and increased mortality in patients with type-2 diabetes have been observed during prior SARS epidemics as well [10, 11], studies conducted during the current COVID-19 pandemic have revealed conflicting results. In a study by Guan et al., the prevalence of type-2 diabetes among the patients who attained primary outcomes defined as admission to intensive care unit, use of mechanical ventilation or mortality was 26.9% as compared to 6.1% in the group who did not [2]. In a large case series from New York including 5700 patients hospitalized with COVID-19, patients with type-2 diabetes who died were more likely to require mechanical ventilation or intensive care unit stay as compared to those who did not have type-2 diabetes [12]. A nationwide observational French CORONADO study [13] involving diabetics hospitalized with COVID-19, surprisingly for many, did not show prior glucose control (determined by hemoglobin A1C) to be a significant determinant of primary outcome defined as death within seven days of admission. In our study, we also noted similar findings where age was a significant predictor of mortality, but neither type-2 diabetes nor prediabetes status predicted mortality (Table 2).

On stratifying our patients further into younger and older age groups (Table 3), we noted no significant difference in mortality among the older patients between the three groups of type-2 diabetes, prediabetes, and nondiabetic groups. However, a significantly higher mortality (27%, *p* 0.025) among the younger type-2 diabetes patients aged less than 55 years was noted as compared with 9% mortality in prediabetes and 12% mortality in the nondiabetic group. These results mean that, in an older patient infected with COVID-19, their glycemic status (type-2 diabetes, prediabetes, or controls) does not predict their mortality and age is a significant predictor of mortality regardless of their diabetes status. However, in younger adults who have lower mortality rates in general as compared to older population, the presence of type-2 diabetes becomes an important determinant of mortality. Similar results were also found in a study by Woolcott and Castilla-Bancayán from Mexico where they found a declining strength of association between diabetes and COVID-19-related mortality with the advancing age [14].

At this time, it is not clearly understood why the mortality in these younger type-2 diabetes patients is higher than those of older type-2 diabetes patients. It is possible that their marginally higher hyperglycemia at the time of admission (285 ± 234 mg/dL vs 257 ± 190 mg/dL, *p* 0.1496) may have played a role. Until further larger studies are conducted to verify our results, it will not be wrong for clinicians to advise their younger type-2 diabetes patients to be mindful of these results and take additional measures to prevent getting infected.

Angiotensin-converting enzyme 2 (ACE-2) has been identified as the site of viral entry into human cells [15]. ACE-2 and its product angiotensin-1-7 play an important anti-inflammatory role under normal circumstances. ACE-2 is present in multiple organs including lungs, renal tubular epithelium, vascular endothelial cells, pancreas, and intestinal endothelium. It hydrolyzes angiotensin-II to yield angiotension-1–7, which plays an important anti-inflammatory and antioxidant role in our bodies. It is postulated that COVID-19 entry into human cells through ACE-2 may lead to imbalance in the angiotensin pathway leading to increase in proinflammatory markers and the subsequent cytokine surge [16]. It is also possible that poor outcomes of patients with COVID-19 in type-2 diabetes is related to unfavorable metabolic milieu of diabetes and its associated comorbid conditions like hypertension, cardiovascular disease, obesity, and advanced age. Insulin resistance and beta-cell dysfunction are the key factors underlying the metabolic derangements noticed in individuals with type-2 diabetes.

Prediabetes is considered as an intermediate state of hyperglycemia where the patients have glucose levels above the normal range but below the diagnostic criteria for diabetes. Patients with prediabetes have similar metabolic derangements, though not to the same extent as seen in type-2 diabetes. As per the International Diabetes Federation, the prevalence of prediabetes in adults in 2017 was 352.1 million [17]. With the current trends towards further rise in the prevalence of prediabetes, we felt it was important to study the effect of COVID-19 in patients with prediabetes. Our study did not see any difference in outcomes in COVID-19-infected patients with prediabetes as compared with the patients without diabetes. To the best of our knowledge, our study is the first in exploring the effect of prediabetes status on outcomes like mortality and need for mechanical ventilation in patients infected with COVID-19.

In our study population with type-2 diabetes, a total of 39 patients (6.2%) were newly diagnosed with type-2 diabetes at the time of admission. The prevalence of undiagnosed type-2 diabetes in our study population is similar to previously reported prevalence of 6.2% among Hispanics and 4% among non-Hispanic Blacks [18]. This small group of 39 patients with newly diagnosed type-2 diabetes had lower mortality rate in comparison with remaining 587 patients of previously diagnosed type-2 diabetes (18% vs 40%, *p* 0.005). These results are in contrast from a study conducted in Italy by Coppelli et al. where they noted higher mortality among newly diagnosed diabetics as compared with previously known diabetics [19]. Requirement for mechanical ventilation was also lower but not statistically significant among these newly diagnosed type-2 diabetes individuals (26% vs 41%, *p* 0.06). Improved survival among these newly diagnosed type-2 diabetes patients as compared with previously known type-2 diabetes patients may have been due to a smaller number of patients and the presence of other confounding variables that can affect mortality like lower mean age of the patients (60.13 ± 15) vs 65.70 ± 60, *p* 0.016), lower mean BMI (29.9 ± +33 vs 33.1 ± 9, *p* 0.02), lower prevalence of hypertension (56% vs 79%, *p* 0.001), greater number of patients treated with remdesivir (5% vs 1%, *p* 0.014), steroids (59% vs 36%, *p* 0.005), and tocilizumab (21% vs 10%, *p* 0.032). On binomial regression analysis, age (OR 1.02, *p* 0.003) and steroid use (OR 0.34, *p* 0.0001) were significant predictors of mortality. Hyperglycemia at the time of admission, which has been previously shown to be a predictor of poor outcomes [20, 21], may have been another reason for higher mortality rates in these patients with previously diagnosed type-2 diabetes infected with COVID-19 (267 ± 206 mg/dL vs 200 ± 90 mg/dL, *p* 0.04). It is also possible that patients with previously diagnosed type-2 diabetes may have had higher prevalence of diabetes microvascular and macrovascular complications which has been shown to be an independent predictor of poor outcomes in patients with COVID-19 [13]. In our patient population, we do not have information about the prevalence of diabetes-related microvascular and macrovascular complications.

This study brings out another important but previously reported finding that admission hyperglycemia is an independent risk factor for worse outcomes and mortality [19, 22]. Our study results are consistent with this important previous finding and show a similar trend of higher mortality among individuals with blood glucose level at admission more than 180 mg/dL in comparison to those with admission glucose levels less than 141 mg/dL, across all 3 categories of controls, prediabetes, and diabetes mellitus.

Our study has several limitations. It is a retrospective study which has its inherent problems due to the retrospective design. Second, our study population mostly comprised of Latinx and African Americans and hence cannot be generalized. Nevertheless, these ethnic groups were not studied in most of the other studies and, hence, our study sheds some light on outcomes in these minority ethnic groups. Third, HbA1c lab values used to categorize patients were not within 3 months for all the patients. A total of 40% patients had HbA1c lab value available from more than 3 months prior to admission. However, the nonavailability of most recent HbA1c in these patients did not lead to misclassification of study patients as electronic medical records were also reviewed before allocating a patient to a particular group. Inaccuracies in documenting the diagnosis of type-2 diabetes and prediabetes in electronic medical record still cannot be circumvented by this approach.

In conclusion, our study results emphasize again the importance of advanced age as a predictor of poor outcome in patients with COVID-19 infection. In patients older than 55 years of age, the status of type-2 diabetes does not influence the mortality rate. However, in younger patients aged less than 55 years, the presence of type-2 diabetes is an important contributor towards mortality. Patients with newly diagnosed type-2 diabetes had better outcomes as compared to previously known type-2 diabetes, likely due to lesser degree of hyperglycemia present at the time of admission, or due to greater number of patients treated with drugs known to influence COVID-19 outcomes, namely, remdesivir, steroids, and tocilizumab. Admission hyperglycemia is associated with higher mortality regardless of diabetes status. Lastly, the presence of prediabetes, which has similar metabolic derangements as type-2 diabetes, did not affect outcomes in patients with COVID-19 infection.

## Figures and Tables

**Table 1:** Comparison of demographic, clinical, and laboratory variables among three groups.

	Control (*n* = 107)	Prediabetes (*n* = 110)	Diabetes mellitus (*n* = 626)	*p* value
Age	58.60 (±17.53)	62.15 (±15.25)	65.36 (±13.96)	**0.001**
BMI	28.07 (±8.06)	29.77 (±8.29)	30.11 (±8.22)	**0.024**

*Outcome*				
Mortality	32 (29.91%)	35 (31.82%)	244 (38.98%)	**0.036**
Mechanical ventilation	33 (30.84%)	34 (30.91%)	250 (39.94%)	**0.027**

*Gender*				0.314
Female	38 (35.51%)	45 (40.91%)	258 (41.21%)	
Male	69 (64.49%)	65 (59.09%)	368 (58.79%)	

*Ethnicity*				0.162
Black	39 (36.45%)	28 (25.45%)	181 (28.91%)	
Caucasian	4 (03.74%)	1 (00.91%)	11 (01.76%)	
Latinx	56 (52.34%)	72 (65.45%)	374 (59.74%)	
Others	8 (07.48%)	9 (08.18%)	60 (09.58%)	

*Clinical presentation*				
Fever	64 (59.81%)	71 (64.55%)	368 (58.79%)	0.57
Cough	69 (64.49%)	69 (62.73%)	379 (60.54%)	0.403
Shortness of breath	67 (62.62%)	74 (67.27%)	408 (65.18%)	0.757
Myalgia	27 (25.23%)	37 (33.64%)	166 (26.52%)	0.774
Headache	8 (07.48%)	9 (08.18%)	46 (07.35%)	0.883
Nausea/vomiting	12 (11.21%)	18 (16.36%)	87 (13.90%)	0.673
Abdominal discomfort	35 (32.71%)	29 (26.36%)	205 (32.75%)	0.644
Diarrhea	15 (14.02%)	13 (11.82%)	85 (13.58%)	0.951

*Comorbid medical conditions*				
HIV/AIDS	18 (16.82%)	8 (07.27%)	40 (06.41%)	**0.001**
Hypertension	52 (48.60%)	63 (57.27%)	482 (77.24%)	**0.001**
Chronic liver disease	2 (01.87%)	1 (00.91%)	7 (01.12%)	0.603
Congestive heart failure	9 (08.41%)	12 (10.91%)	75 (12.02%)	0.28
Coronary artery disease	6 (05.61%)	9 (08.18%)	97 (15.54%)	**0.001**
Chronic kidney disease	8 (07.48%)	7 (06.36%)	74 (11.88%)	0.07

*SARS-CoV-*2 *management*				
Hydroxychloroquine	74 (69.16%)	86 (78.18%)	498 (79.55%)	**0.025**
Antiviral agents	13 (12.15%)	14 (12.73%)	52 (08.31%)	0.104
Antibiotics	100 (93.46%)	104 (94.55%)	600 (95.85%)	0.241
Tamiflu	90 (84.11%)	96 (87.27%)	565 (90.26%)	**0.046**
Remdesivir	0 (00.00%)	2 (01.82%)	7 (01.12%)	0.486
Anticonvalescent plasma	1 (00.93%)	6 (05.45%)	20 (03.19%)	0.486
Steroids	45 (42.06%)	47 (42.73%)	237 (37.86%)	0.288
Therapeutic anticoagulation	60 (56.07%)	77 (70.00%)	394 (62.94%)	0.471
Tocilizumab	5 (04.67%)	9 (08.18%)	65 (10.38%)	0.055

*Laboratory parameters*				
Serum creatinine (mg/dL)	2.57 (4.60)	1.73 (±3.13)	2.20 (±2.59)	0.607
White blood cell count (k/*μ*L)	8.65 (±8.64)	8.17 (±3.89)	8.93 (±7.00)	0.476
Absolute neutrophil count (k/*μ*L)	6.32 (±3.47)	6.60 (±3.51)	7.14 (±4.24)	**0.03**
Absolute lymphocyte count (k/*μ*L)	1.72 (±8.09)	0.98 (±0.66)	1.17 (±5.20)	0.458
ANC/ALC ratio	10.23 (±11.90)	9.41 (±7.74)	10.20 (±11.28)	0.83
D-dimer (ng/mL)	385.77 (±233.88)	399.52 (±223.72)	415.71 (±235.91)	0.223
Serum lactate dehydrogenase (U/L)	447.54 (±234.92)	463.17 (±177.18)	447.62 (±212.98)	0.813
C-reactive protein (mg/L)	109.18 (±110.48)	130.33 (±88.31)	144.31 (±113.27)	**0.005**
Serum ferritin (ng/mL)	371.06 (±267.68)	390.66 (±254.71)	390.34 (±259.06)	0.569

**Table 2:** Binomial regression analysis for mortality.

	Odds ratio	*p*-value
Age	1.03 (1.02–1.05)	**0.001**
Control	0.00 (0.00–0.00)	0.83
Prediabetes	0.84 (0.47–1.49)	0.55
Diabetes mellitus	0.94 (0.57–1.55)	0.82
BMI	1.02 (1.00–1.04)	0.12
HIV/AIDS	0.36 (0.20–0.65)	**0.001**
Hypertension	0.91 (0.60–1.37)	0.64
CAD	0.69 (0.43–1.11)	0.13
Absolute neutrophil count	1.02 (0.97–1.06)	0.45
CRP	1.00 (1.00–1.01)	**0.001**
Hydroxychloroquine	0.48 (0.31–0.76)	**0.002**
Tamiflu	0.77 (0.41–1.42)	0.40

**Table 3:** Binomial regression analysis for mechanical ventilation use.

	Odds ratio	*p* value
Age	1.01 (1.00–1.02)	0.210
Control	0.00 (0.00–0.00)	0.629
Prediabetes	0.86 (0.49–1.49)	0.584
Diabetes mellitus	0.80 (0.49–1.32)	0.388
BMI	1.03 (1.01–1.05)	**0.013**
HIV/AIDS	0.72 (0.40–1.31)	0.280
Hypertension	0.80 (0.54–1.19)	0.279
CAD	1.02 (0.63–1.65)	0.931
Absolute neutrophil count	0.98 (0.94–1.02)	0.391
CRP	1.00 (1.00–1.01)	≤0.001
Hydroxychloroquine	0.28 (0.17–0.46)	≤0.001
Tamiflu	0.70 (0.37–1.31)	0.260

**Table 4:** Comparison of mortality based on the age group.

	Survived, *n* (%)	Mortality, *n* (%)	*p* value
*Age <*55			**0.025**
Nondiabetic (*n* 40)	35 (88%)	5 (12%)	
Prediabetes (*n* 33)	30 (91%)	3 (9%)	
Diabetes mellitus (*n* 137)	100 (73%)	37 (27%)	

*Age >*55			0.948
Nondiabetic (*n* 67)	40 (60%)	27 (40%)	
Prediabetes (*n* 77)	45 (59%)	32 (41%)	
Diabetes mellitus (*n* 489)	282 (58%)	207 (42%)	

**Table 5:** Comparison of mechanical ventilation use based on the age group.

	Survived, *n* (%)	Mortality, *n* (%)	*p* value
*Age <*55			0.109
Nondiabetic (*n* 40)	32 (80%)	8 (20%)	
Prediabetes (*n* 33)	25 (76%)	8 (24%)	
Diabetes mellitus (*n* 137)	88 (64%)	49 (36%)	

*Age >*55			0.43
Nondiabetic (*n* 67)	40 (60%)	27 (40%)	
Prediabetes (*n* 77)	51 (66%)	26 (34%)	
Diabetes mellitus (*n* 489)	288 (59%)	201 (41%)	

**Table 6:** Comparison of demographic, clinical, and laboratory variables among the previously and newly diagnosed type-2 diabetes patients.

	Previously diagnosed diabetes mellitus (*n* = 587)	Newly diagnosed diabetes mellitus (*n* = 39)	
Age	65.70 (±60.13)	60.13 (±15.02)	**0.016**
BMI	29.91 (±33.05)	33.05 (±9.17)	**0.021**
Mortality	237 (40.37%)	7 (17.95%)	**0.005**
Mechanical ventilation	240 (40.89%)	10 (25.64%)	0.06

*Gender*			
Female	243 (41.40%)	15 (38.46%)	
Male	344 (58.60%)	24 (61.54%)	

*Ethnicity*			**0.013**
Black	174 (29.64%)	7 (17.95%)	
Caucasian	11 (01.87%)	0 (00.00%)	
Hispanic	350 (59.63%)	24 (61.54%)	
Others	52 (08.86%)	8 (20.51%)	

*Clinical presentation*			
Fever	342 (58.26%)	26 (66.67%)	0.303
Cough	350 (59.63%)	29 (74.36%)	0.068
Shortness of breath	377 (64.22%)	31 (79.49%)	0.053
Myalgia	155 (26.41%)	11 (28.21%)	0.806
Headache	40 (06.81%)	6 (15.38%)	**0.047**
Nausea/vomiting	82 (13.97%)	5 (12.82%)	0.841
Abdominal discomfort	198 (33.73%)	7 (17.95%)	**0.042**
Diarrhea	78 (13.29%)	7 (17.95%)	0.411

*Comorbid medical conditions*			
HIV/AIDS	39 (06.67%)	1 (02.56%)	0.312
Hypertension	460 (78.63%)	22 (56.41%)	**0.001**
Congestive heart failure	72 (12.31%)	3 (07.69%)	0.392
Coronary artery disease	92 (15.73%)	5 (12.82%)	0.628
Chronic kidney disease	72 (12.33%)	2 (05.13%)	0.179

*SARS-CoV-*2 *management*			
Hydroxychloroquine	464 (79.05%)	34 (87.18%)	0.223
Antiviral agents	45 (7.67%)	7 (17.95%)	**0.024**
Antibiotics	561 (95.57%)	39 (100.00%)	0.18
Tamiflu	58 (9.88%)	3 (7.69%)	0.656
Remdesivir	5 (0.85%)	2 (5.13%)	**0.014**
Anticonvalescent plasma	17 (02.90%)	3 (07.69%)	0.09
Steroids	214 (36.46%)	23 (58.97%)	**0.005**
Therapeutic anticoagulation	365 (62.18%)	29 (74.36%)	0.128
Tocilizumab	57 (09.71%)	8 (20.51%)	**0.032**

*Laboratory parameters*			
Serum creatinine	2.25 (±1.46)	1.46 (±1.51)	0.063
White blood cell count	8.93 (±8.94)	8.94 (±3.76)	0.993
Absolute neutrophil count	7.13 (±7.41)	7.41 (±3.60)	0.691
Absolute lymphocyte count	1.19 (±0.90)	0.90 (±0.45)	0.737
ANCALC ratio	10.19 (±10.29)	10.29 (±8.93)	0.959
D-dimer	414.19 (±435.77)	435.77 (±252.31)	0.582
Serum lactate dehydrogenase	446.99 (±456.26)	456.26 (±189.55)	0.793
C-reactive protein	141.87 (±177.39)	177.39 (±134.83)	0.059

**Table 7:** Binomial regression for mortality in the patients with diabetes mellitus.

	Odds ratio	*p* value
Previously diagnosed diabetes mellitus	3.50 (1.45–8.46)	**0.005**
Age	1.02 (1.01–1.04)	**0.003**
BMI	1.00 (0.97–1.02)	0.751
Black	0.00 (0.00–0.00)	0.224
Caucasian	1.04 (0.54–2.01)	0.902
Hispanic	0.95 (0.22–4.10)	0.945
Others	1.50 (0.81–2.77)	0.200
Hypertension	0.77 (0.49–1.21)	0.260
Antiviral medication	1.11 (0.59–2.09)	0.752
Remdesivir	0.36 (0.06–2.18)	0.265
Anticonvalescent plasma	2.07 (0.64–6.71)	0.224
Steroids	0.34 (0.24–0.50)	≤0.001
Tocilizumab	1.36 (0.69–2.65)	0.374

**Table 8:** Mortality according to admission blood glucose level.

Admission blood glucose level (mg/dL)	Controls (normoglycemic)	Prediabetes	Diabetes mellitus
<140	28.2% (24/85)	28.4% (25/88)	28.5% (50/175)
141–180	23.1% (3/13)	41.2% (7/17)	43.2% (48/111)
>180	55.5% (5/9)	60% (3/5)	42.9% (146/340)

## Data Availability

The tables and figures used to support the findings of this study are included within the article.
